# Supplementary material for: Heritability of growth and leaf loss compensation in a long-lived tropical understorey palm
Source: PLoS One. 2019 May 2;14(5):e0209631. doi: 10.1371/journal.pone.0209631 (PMC6497226; doi:10.1371/journal.pone.0209631)
Supplement: S1 File — Details on methods of the construction of an allometric model for estimation of biomass per plant part of seedlings of 6 months of age. (DOCX) [file pone.0209631.s001.docx]

**Supporting Information 1** *Allometric model*

To be able to relate non-destructive measurements of seedlings at six months of age to seedling biomass (per plant part) and leaf area, we constructed an allometric model based on regression results.

Six months before the start of the main experiment, we planted *Chamaedorea elegans* seeds obtained from a Dutch commercial grower (Aardam Planten), and we grew the seedlings under the same experimental conditions as the main experiment. We destructively harvested 61 of these seedlings at six months of age. We measured seedling stem length and diameter, of all leaves leaf width, lamina length, rachis length, rachis diameter, leaflet width, number of leaflets, and length of the unopened leaf. Furthermore, we determined biomass per plant part, and leaf area, where we followed the same procedures as in the main experiment. We also visually inspected seed remains, and found all remaining seed coats to be empty, suggesting seed reserves were depleted.

For each response variable, we analysed a full model in which all relevant explanatory variables and likely non-linear terms were included and selected from this the best model (based on AIC and degrees of freedom) using the dredge function of the MuMIn package in R [1]. We constructed separate models for leaf area, leaf mass, rachis mass, the mass of the unopened leaf, stem mass and root mass. The resulting statistical models are shown in the table below. These models were used to, from, at the start of the experiment non-destructively measured plant size parameters, estimate biomass per plant part and leaf area of seedlings of six months of age.

|  |  | **Estimate** | **P** | **R^2^** |
| --- | --- | --- | --- | --- |
| **Leaf area** | **Intercept** | 0.491 | 0.663 | 0.957 |
|  | **Leaf length^2^** | 0.00748 | <0.001 |  |
|  | **Rachis diameter^2^** | 2.16 | <0.001 |  |
|  | **Leaf width^2^** | 6.66E-04 | <0.001 |  |
| **Leaf mass** | **Intercept** | 6.11E-03 | 0.0541 | 0.961 |
|  | **Leaf length^2^** | 2.83E-05 | <0.001 |  |
|  | **Leaf width^2^** | 3.23E-06 | <0.001 |  |
| **Rachis mass** | **Intercept** | 7.04E-03 | 0.11 | 0.914 |
|  | **Rachis length** | 3.73E-04 | <0.001 |  |
|  | **Leaf length** | -6.11E-04 | <0.001 |  |
|  | **Leaf length^2^** | 6.54E-06 | 0.00982 |  |
|  | **Rachis diameter^2^** | 2.88E-03 | <0.001 |  |
|  | **Leaf width** | -3.01E-04 | 0.0204 |  |
|  | **Leaf width^2^** | 2.21E-06 | 0.0238 |  |
| **Unopened leaf mass** | **Intercept** | 1.93E-03 | 0.406 | 0.922 |
|  | **Unopened leaf length^2^** | 1.03E-05 | <0.001 |  |
| **Stem mass** | **Intercept** | 5.33E-02 | 0.00622 | 0.973 |
|  | **Stem diameter** | -4.70E-02 | <0.001 |  |
|  | **Stem length^2^** | 2.61E-05 | <0.001 |  |
|  | **Stem diameter^2^** | 1.21E-02 | <0.001 |  |
| **Root mass** | **Intercept** | 7.70E-02 | 0.00109 | 0.972 |
|  | **Stem length** | -9.03E-03 | <0.001 |  |
|  | **Stem length^2^** | 1.88E-04 | <0.001 |  |
|  | **Stem diameter^2^** | 7.04E-03 | <0.001 |  |

**References**

1. Barton K. MuMIn: Multi-Model Inference. R package version 1.13.4. 2015.
