# Supplementary material for: Heritability of growth and leaf loss compensation in a long-lived tropical understorey palm
Source: PLoS One. 2019 May 2;14(5):e0209631. doi: 10.1371/journal.pone.0209631 (PMC6497226; doi:10.1371/journal.pone.0209631)
Supplement: S2 File — Details on methods of the construction and adaptation of an iterative growth model for estimation of daily individual seedling NAR, flam and γ. (DOCX) [file pone.0209631.s002.docx]

**Supporting Information 2** *Iterative growth model.*

The input of the iterative growth model presented by Anten and Ackerly [1] is biomass, leaf mass, and leaf area at the beginning and end of the experiment, and leaf loss (mass and area, and time of removal) during the experiment. We, however, did not measure leaf loss directly but assumed this to be two third of existing leaf mass. To allow for this, we adjusted the Anten and Ackerly [1] model. Mathematically this model can be described in the following way. As in Anten and Ackerly [1], daily changes in total plant mass (W), leaf mass (L) and leaf area (A), from time=t to time=t+1 are:

*W_t+1_=W_t_+G_t_-W_loss,t_*  eqn S1a

*L_t+1_=L_t_+f_lam_*G_t_-L_loss,t_* eqn S1b

*A_t+1_=A_t_+γ*SLA_t_*f*G_t_-A_loss,t_* eqn S1c

In which

*G_t_=A_t_*NAR*  eqn S1d

and in which *f_lam_* is the fraction of newly assimilated mass that is allocated to lamina growth, γ the proportional difference between the specific leaf area (SLA) of newly produced leaves and the current mean SLA of the plant, and NAR the net assimilation rate. We extended this model by describing the changes in plant weight and leaf area on days that defoliation occurs as:

$W_{loss,t}=L_{loss,t}=\frac{2}{3}\left( L_{t}-\frac{1}{2}\sum_{i=0}^{t-1} L_{loss,i} \right)$ eqn S2a

$A_{loss,t}=\frac{2}{3}\left( A_{t}-\frac{1}{2}\sum_{i=0}^{t-1} A_{loss,i} \right)$ eqn S2b

where i indicates the different days. The total loss up to time t-1 multiplied by ½ is remaining leaf area at time t-1. L_t_ – remaining leaf area is newly produced leaf area. In this way we estimated daily NAR, f_lam_ and γ for each individual, using the R function nls, using the port algorithm and with realistic start values and boundaries provided (estimated from data and literature). We applied the extra restriction that the total leaf loss must equal estimated leaf loss from direct biomass measurements. Using the estimated values of NAR, f_lam_ and γ, RGR was calculated per iteration step, and then averaged. NAR, f_lam_ and γ estimations were also averaged. R-script of this model is available upon request.

**References**

1. Anten NPR, Ackerly DD. A new method of growth analysis for plants that experience periodic losses of leaf mass. Functional Ecology. 2001;15(6):804-11. PubMed PMID: 267.
